# Case Report: Findings Suggestive of Paraclinical Progressive Multifocal Leukoencephalopathy and Lung Cancer-Derived Brain Metastases in an MS Patient Treated With Fingolimod

**DOI:** 10.3389/fneur.2021.561158

**Published:** 2021-02-03

**Authors:** Fabian Maass, Philipp von Gottberg, Jonas Franz, Christine Stadelmann, Mathias Bähr, Martin S. Weber

**Affiliations:** ^1^Department of Neurology, University Medical Center Göttingen, Göttingen, Germany; ^2^Department of Neuroradiology, University Medical Center Göttingen, Göttingen, Germany; ^3^Institute of Neuropathology, University Medical Center Göttingen, Göttingen, Germany

**Keywords:** multiple sclerosis, fingolimod (FTY720), lung cancer, RRMS (relapsing-Remitting MS), PML–progressive multifocal leucoencephalopathy

## Abstract

Fingolimod represents a highly effective disease-modifying drug in patients with active relapsing-remitting multiple sclerosis (RRMS). Its immunosuppressive effects can mediate adverse events like increased risk of cancer development or appearance of opportunistic infections. Progressive multifocal leukoencephalopathy (PML)–representing a severe opportunistic infection–has been only infrequently described during Fingolimod treatment. Here, we present a case of a 63-year-old women with pre-diagnosed RRMS who presented with new multiple cerebral lesions in a routine MRI scan, also including a tumefactive lesion in the left parietal lobe, eventually leading to the diagnosis of brain metastases derived by an adenocarcinoma of the lung. Additionally, a JCV-DNA-PCR in the cerebrospinal fluid revealed positive results, corresponding to a paraclinical progressive multifocal leukoencephalopathy. In conclusion, adverse events potentially associated with immunosuppression can occur during Fingolimod treatment. In this context, the occurrence of cancer and opportunistic infections should be carefully monitored. Here, we report a case in which JCV-DNA-PCR in the cerebrospinal fluid suggests asymptomatic PML and simultaneously lung cancer brain metastases developed. While it is rather unlikely that either event occurred as an adverse event of fingolimod treatment, a contributing effect cannot be formally excluded.

## Introduction

Fingolimod (FTY720)—a sphingosine-1-phosphate-receptor modulator–is now commonly used in highly active relapsing-remitting multiple sclerosis (RRMS), due to its proven ability to prevent potential relapses (1). Despite the clear evidence for disease modifying effects, adverse events caused by the immunosuppressive effect potentially resulting in an increased risk of oncogenesis and appearance of opportunistic infections must be considered (2).

## Case Presentation

A 63-year-old caucasian women with a prior diagnosis of MS (RRMS; initial diagnosis ~12 years ago) was admitted to our department because of a suspicious MRI scan, regularly arranged by her neurologist. This scan was now showing multiple new gray and white matter lesions. She had no new subjective complaints, still showing a relatively mild course of her disease (EDSS 1.5) with a slight cerebellar ataxia and slight sensory symptoms. At the age of 56 she was switched to fingolimod and had therapeutic attempts with different types of interferons and glatiramer acetate beforehand. Of note, the patient had never been treated with natalizumab.

**Figure 1:**
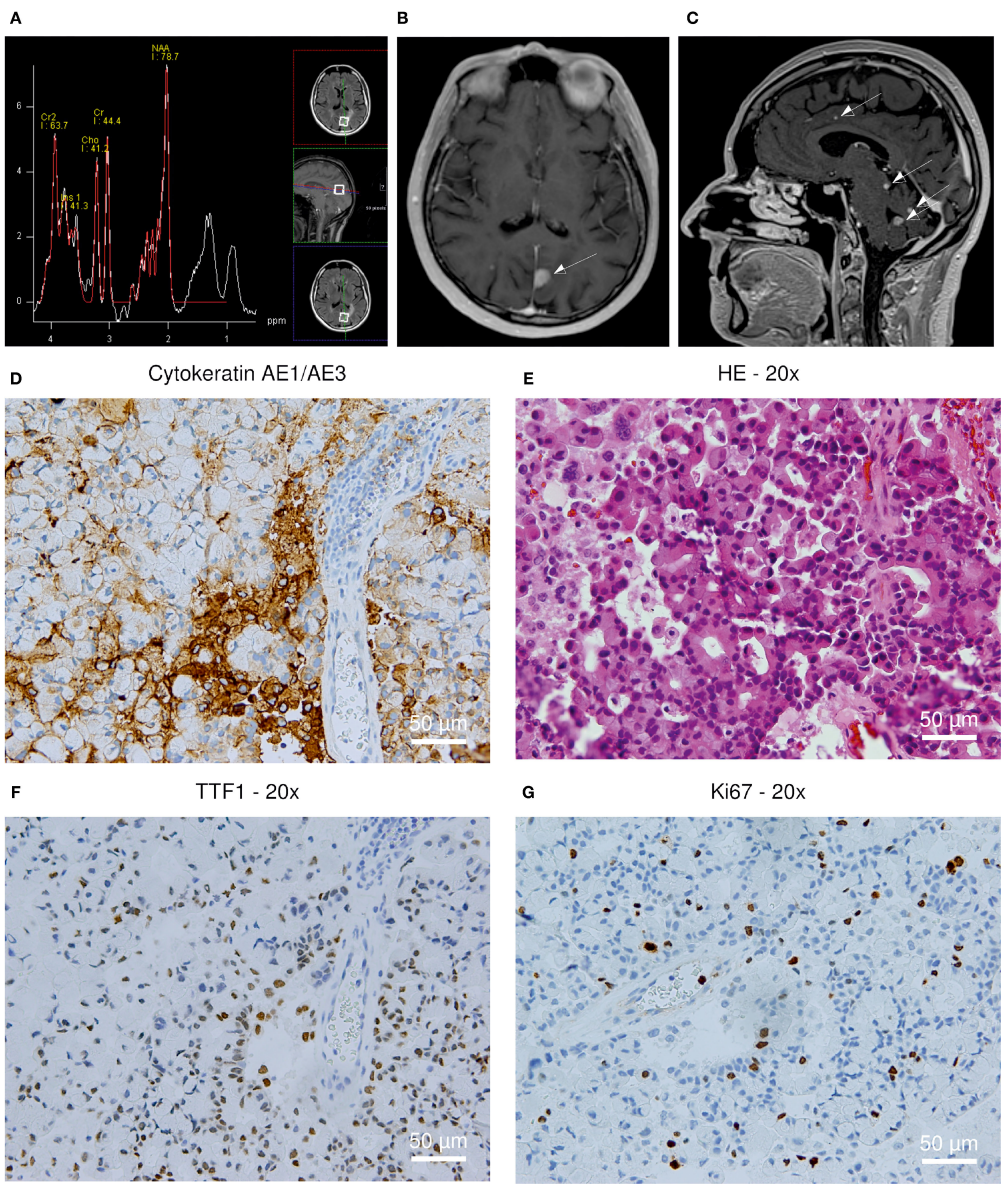
**(A–C)** MRI scan revealing multiple T2/FLAIR hyperintensities; **(D–G)** Histopathological analysis of the brain biopsy. **(A)** MRI spectroscopy showing a decreased N-acetylaspartate (NAA) peak. **(B)** New prominent tumefactive lesion in the left parietal lobe **(C)** New disseminated supra- and infratentorial hyperintensities **(D)** Cytokeratin AE1/3+ positivity; 200x **(E)** Hematoxylin-eosin staining; 200x **(F)** TTF1+ positivity; 200x **(G)** Ki67 staining; 200x.

Directly after admission, we performed a new MRI scan applying a gadolinium-based contrast agent now showing disseminated supra- and infratentorial T2/FLAIR hyperintensities, predominantly in the gray matter, also including a prominent tumefactive lesion in the left parietal lobe (Figures 1B,C). The MRI presentation itself did not allow to assign these new lesions to PML, MS activity or the development of metastases. MRI spectroscopy showed decreased N-acetylaspartate (NAA) indicating a neurodestructive, non-primarily-CNS-derived and thus malign process (Figure 1A).

A peripheral immune cell quantification revealed a reduced white blood cell count (3,500/μl) with lymphopenia (318/μl) and a reduction in CD3+/CD4+ (22/μl) and CD19+ cells (7/μl), as well as normal numbers of CD56+ cells (146/μl).

Analyses of the cerebrospinal fluid (CSF) initially revealed typical RRMS-associated findings including a normal cell count, increased albumin levels and the detection of CSF-specific oligoclonal bands. An additional JCV-(John Cunningham virus)-DNA-PCR yielded the detection of 39 copies per mL, consistent with the laboratory finding of a paraclinical progressive multifocal leukoencephalopathy (PML).

Furthermore, we quantified carcinoembryonic antigen (CEA) in the CSF, revealing markedly increased levels, therefore pointing to probable carcinoma-derived metastases. A subsequent thoracic CT scan showed a suspicious pulmonal lesion (diameter of 2.9 cm) in the right apical lobe without radiological proof of lymphadenopathy. The patient was non-smoker and the family history was negative concerning the occurrence of lung cancer. No other risk factors have been reported by the patient.

To determine its origin, a stereotactic biopsy of the tumefactive lesion in the left parietal lobe was performed, revealing TTF1+, CK7+, AE1/3+ tissue, consistent with a metastasis derived from a bronchial adenocarcinoma. Additional immunohistochemistry analyses revealed negative results for SV40 and P53, therefore excluding an overlap with PML-associated changes in the region of the biopsy (Figures 1D–G).

Consequently, fingolimod treatment was immediately discontinued after positive JCV-DNA-PCR. Finally, the patient was admitted to the department of oncology for further diagnostic and interdisciplinary therapy initiation.

## Discussion

Here, we report a case which highlights multiple challenges which can occur in active treatment of RRMS.

Occurrence of new cerebral lesions during fingolimod treatment must lead to the differential diagnosis of (1) an insufficient treatment effect associated with new disease activity or (2) a possible intracerebral adverse event.

With an estimated risk is of 0.069 per 1,000 patients, PML caused by JCV is quite rare during fingolimod treatment (3) and the MRI lesions in our case did not present with typical associated characteristics such as the prominent involvement of subcortical U-fibers (4). Nevertheless, low copy numbers of JCV-DNA could be detected in the CSF, potentially reflecting a subclinical stage of a developing PML. Such stages are rarely seen, because patients usually do not receive lumbar puncture in absence of new clinical signs and await to be better understood in the future. Limiting, an additional JCV-PCR on the biopsy material was not performed in this case. Verification of JCV-DNA in the brain biopsy would have reinforced the hypothesis of a potential subclinical PML, reflected by a low copy number of JCV-DNA in the CSF.

Because of the tumefactive characteristics of the lesion, CEA was quantified in the CSF. Increased levels can be detected especially in the presence of leptomeningeal infiltration by carcinoma but to a lesser extent also in the case of intraparenchymal infiltration (5). Due to similar molecular size, intrathecal CEA synthesis can be reliable assessed applying an IgA diagram (6).

Different types of skin cancer but also breast cancer were reported during fingolimod treatment (2). Lung cancer was less frequent and paradoxically, fingolimod is discussed to mediate lung tumor suppression by alteration of protein phosphatase 2A (7). Therefore, any causal relationship between lung cancer and fingolimod treatment in our current case is rather uncertain. The estimated incidence rate for invasive cancer development during Fingolimod treatment has been reported with 44.0 per 10,000 person-years, discussed to present an slightly increased risk compared to the general population with an incidence rate of 31.0 (8). Therefore, the usage of repeated preventive diagnostics might be not reasonable with Fingolimod treatment for patients with low risk of lung cancer development but should be considered in patients with high risk (e.g., smoker, positive family history).

Reports of concomitant occurrence of PML and primary central nervous system malignancies (e.g., primary CNS lymphoma) can be found in literature (9). Interestingly, JC-virus can also present with oncogenic potential in complete absence of a PML. Initially reported in animal models, JVC can induce tumors of glial origin. In humans, JCV seems to be especially associated with medulloblastomas among other primary brain tumors, e.g., glioblastoma multiforme and primary CNS lymphomas, as reviewed by Ahye et al. (10).

## Conclusion

This case highlights that fingolimod represents a highly effective disease-modifying drug in prevention of MS relapses and accumulating disability, but that the concomitant occurrence of rare, but possibly severe adverse events such as neoplasia or opportunistic infections must be monitored. Furthermore, our case exemplifies that PML during fingolimod treatment can present with very mild, even isolated paraclinical disease course, and may accordingly be more frequent than reported.

## Data Availability Statement

The original contributions presented in the study are included in the article/supplementary material, further inquiries can be directed to the corresponding author/s.

## Ethics Statement

Written informed consent was obtained from the individual(s) for the publication of any potentially identifiable images or data included in this article.

## Author Contributions

FM, MW, PG, JF, CS, and MB: data acquisition, analysis, and revising the manuscript. PG, JF, and FM: figure design. FM and MW: drafting the manuscript. All authors read and approved the final manuscript.

## Conflict of Interest

The authors declare that the research was conducted in the absence of any commercial or financial relationships that could be construed as a potential conflict of interest.
